# The Co-Injection of Somatic Cells with Embryonic Stem Cells Affects Teratoma Formation and the Properties of Teratoma-Derived Stem Cell-Like Cells

**DOI:** 10.1371/journal.pone.0105975

**Published:** 2014-09-02

**Authors:** Seung Pyo Gong, Boyun Kim, Hyo Sook Kwon, Woo Sub Yang, Jae-Wook Jeong, Jiyeon Ahn, Jeong Mook Lim

**Affiliations:** 1 Major in Biomodulation and Department of Agricultural Biotechnology, Seoul National University, Seoul, Korea; 2 Department of Marine Bio-materials and Aquaculture, Pukyong National University, Busan, Korea; 3 Department of Obstetrics, Gynecology & Reproductive Biology, College of Human Medicine, Michigan State University, Grand Rapids, Michigan, United States of America; 4 Research Institute for Agriculture and Life Science, Seoul, Korea; University of Kansas Medical Center, United States of America

## Abstract

The aim of this study was to assess the biological reactions triggered by stem cell transplantation related to phenotypic alteration, host-to-cell response, chromosomal stability, transcriptional alteration, and stem cell-like cell re-expansion. B6CBAF1 mouse embryonic stem cells (ESCs) were injected subcutaneously into homologous or heterologous (B6D2F1) recipients, and heterologous injections were performed with or without co-injection of B6D2F1 fetal fibroblasts. All homologous injections resulted in teratoma formation, whereas a sharp decrease in formation was detected after heterologous injection (100 vs. 14%; *p*<0.05). The co-injection of somatic cells in heterologous injections enhanced teratoma formation significantly (14 vs. 75%; *p*<0.05). Next, ESC-like cell colonies with the same genotype as parental ESCs were formed by culturing teratoma-dissociated cells. Compared with parental ESCs, teratoma-derived ESC-like cells exhibited significantly increased aneuploidy, regardless of homologous or heterologous injections. Repopulation of the parental ESCs was the main factor that induced chromosomal instability, whereas the co-injection of somatic cells did not restore chromosomal normality. Different genes were expressed in the parental ESCs and teratoma-derived ESC-like cells; the difference was larger with parental vs. heterologous than parental vs. homologous co-injections. The co-injection of somatic cells decreased this difference further. In conclusion, the host-to-cell interactions triggered by ESC transplantation could be modulated by co-injection with somatic cells. A mouse model using homologous or heterologous transplantation of stem cells could help monitor cell adaptability and gene expression after injection.

## Introduction

The transplantation of stem cells into living recipients causes several biological reactions in both the transplanted cells and recipient. First, the injected cells might trigger host-to-cell immune responses; the regulation of this reaction is essential for successful migration and homing after transplantation. Second, the injected stem cells might respond unexpectedly to the unfamiliar microenvironment. Several reports have suggested that various cellular changes occur after exposure to different microenvironments [1]–[4], and that the host microenvironment stimulates cellular plasticity to generate both normal and cancer cell progenitors [5], [6]. Genetic instability and an altered phenotype are also common after transplantation [7], [8]. Understanding all the events that accompany injection will contribute significantly to the development of clinically useful transplantation technologies and novel strategies for regenerative medicine.

A suitable model is required to monitor the wide spectrum of transplantation-related cellular events. In this study, we introduced a model of stem cell and somatic cell co-injection and then monitored the donor-to-stem cell interactions by modulating donor-recipient homogeneity and injection methods. We used this model to examine the various biological reactions triggered by stem cell transplantation, including phenotypic changes, host-to-cell responses, chromosomal stability, transcriptional alteration, and stem cell-like cell re-expansion in neoplastic tissues. We employed F1 mouse embryonic stem cell (ESC) donors, homologous or heterologous ESC recipients, and somatic cells homologous to the recipients to provide different environments for ESC transplantation.

## Materials and Methods

### Experimental design

A total of 1×10^7^ B6CBAF1 ESCs in 100 µl phosphate-buffered saline (PBS; GIBCO Invitrogen, Grand Island, NY) were injected subcutaneously into four homologous or seven heterologous (B6D2F1) recipients. For heterologous co-injections, somatic cells homologous to the recipients were co-injected with B6CBAF1 ESCs (a somatic cell: ESC ratio of 1∶4; 1×10^7^ cells/100 µl PBS) into four heterologous (B6D2F1) recipients. Mouse fetal fibroblasts (MFFs) were employed as the homologous somatic cells. As a control, vehicle (100 µl PBS only) or somatic cells alone (1×10^7^ cells in 100 µl PBS) were injected into two homologous recipients. The transformation of the injected cells was monitored 5 weeks after injection by biopsy after surgical collection, and the neoplastic tissues were subsequently cultured *in vitro* following enzymatic dissociation. Phenotypic observations were made, and the expression of specific markers was monitored. If the neoplastic tissue cultures yielded ESC-like cells, differentiation activity *in vitro* and chromosome normality were also evaluated. After the various co-injections, whole genome expression of parental ESCs was compared with that of the repopulated ESC-like cells using DNA microarrays.

### Experimental animals

Two F1 hybrid mice were produced by mating female C57BL/6 with male DBA2 or CBA/ca mice and were maintained in the Laboratory of Stem Cell and Bioevaluation at Seoul National University under controlled lighting (14∶10-hour light-dark cycle), temperature (20–22°C) and humidity (40–60%). All procedures for animal management, breeding, and surgery followed the standard protocols of Seoul National University, Korea. The experimental samples were managed appropriately, and quality control of the laboratory facility and equipment were performed. The Institutional Animal Care and Use Committee Review Board at Seoul National University approved the research proposal (approval number: SNU-070423-4), including permission for all methods used for animal treatment and euthanasia based on regulation using the 3Rs (replacement, reduction, and refinement). All cell injection procedures were performed after tranquilization through intraperitoneal injection of 0.25% Avertin (2, 2,–tribromoethyl alcohol, Sigma–Aldrich, St. Louis, MO) at 0.01 ml per gram of body weight. Those cell recipients with formation of neoplastic masses in their abdomen were euthanized by cervical dislocation, and teratoma tissues were isolated. All efforts were made to minimize suffering.

### Preparation and culture of ESCs and somatic cells

ESCs and somatic cells were used as the donor cells for co-injection. B6CBAF1 ESCs used in this study were established in our previous study via the expansion of inner cell mass of the blastocyst which was derived from mating female C57BL/6 and male CBA/ca mice [9]. To derive MFFs, 13.5-day post-coitus fetuses from the B6D2F1 and ICR strains were sacrificed, and their visceral organs, heads, and extremities were removed under a microscope. The MFFs were then collected from the remaining tissue after dissociation using 0.04% trypsin-EDTA (GIBCO Invitrogen). ESCs were cultured on a mitotically-inactivated ICR MFF monolayer treated with 10 µg/ml mitomycin C (Sigma-Aldrich) in Dulbecco's modified eagle's medium (DMEM; GIBCO Invitrogen) containing 2 mM L-glutamine (Sigma-Aldrich), 0.1 mM β-mercaptoethanol (GIBCO Invitrogen), 1% (v/v) nonessential amino acids (GIBCO Invitrogen), 1% (v/v) penicillin/streptomycin (GIBCO Invitrogen), 15% FBS, and 1,000 units/ml mouse leukemia inhibitory factor (LIF; Chemicon, Temecula, CA). The somatic cells were cultured in the same basal medium supplemented with 10% FBS and 1% (v/v) penicillin/streptomycin.

### Co-injection and derivation of cell lines from teratomas

Prior to use in allografts, the ESCs were characterized by monitoring stemness-specific gene and protein expression, karyotypes, and differentiation activity. A total of 1×10^7^ cells (somatic cell: ESC ratio of 1∶4) were injected subcutaneously into B6CBAF1 or B6D2F1 hybrid mice. After 5 weeks, the teratomas were retrieved and dissociated in DMEM containing 0.25% trypsin/EDTA and 750 units/ml collagenase type I (Sigma-Aldrich) at 37°C for 30 min. The cells that dissociated from teratomas were then cultured on a mitotically inactivated ICR MFF monolayer in ESC culture medium containing 2,000 units/ml mouse LIF.

### Characterization of parental ESCs and re-expanded ESC-like cells

To characterize the expression of stem cell-specific markers, after the 20^th^ subculture, cells were washed in PBS lacking Ca^2+^ and Mg^2+^ and fixed in 4% (v/v) formaldehyde (Sigma-Aldrich) at room temperature for 10 min. After two washes with PBS, the samples were immunostained with antibodies against Oct-4 (Santa Cruz Biotechnology, Santa Cruz, CA) for 1 h at room temperature. To detect antigen/antibody complexes, the samples were incubated with FITC-conjugated goat anti-mouse IgM secondary antibodies (Molecular Probes, Eugene, OR) for 1 h at room temperature. The nuclei were counterstained using DAPI (Sigma-Aldrich). The stained images were captured using laser scanning confocal microscopy (Bio-Rad, Hemel Hempstead, UK). In addition, the alkaline phosphatase activity of the samples was assessed using Fast Red TR/naphthol AS-MX phosphate (Sigma-Aldrich).

Reverse transcription (RT)-PCR was performed to identify stem cell-specific gene expression in ESCs and ESC-like cells. Total RNA was extracted from the samples using an RNeasy Plus Mini Kit (Qiagen, Valencia, CA) according to the manufacturer's instructions. The cDNAs were synthesized using the SuperScript III First-Strand Synthesis system (Invitrogen) and were PCR-amplified using specific primers. The PCR products were size-fractionated using 1.2% agarose gel electrophoresis (Bioneer, Seoul, South Korea) and visualized using ethidium bromide (EtBr) staining (Bioneer). The sequences of all primers used for PCR amplifications are shown in Table 1.

**Table 1:** Primer sequences

Genes	Accession Number	Primer sequence		Product Size (bp)
		Sense (5′>3′)	Anti-sense (5′>3′)	
*β-actin* (RT)	NM_007393	ACCGTGAAAAGATGACCCAG	TCTCAGCTGTGGTGGTGAAG	273
*β-actin* (R-T)	NM_007393	TACCACAGGCATTGTGATGG	TCTTTGATGTCACGCACGATT	201
*Oct-4* (RT)	NM_013633	GAAGCCCTCCCTACAGCAGA	CAGAGCAGTGACGGGAACAG	297
*Nanog* (RT)	NM_028016	CCCCACAAGCCTTGGAATTA	TCAAATCCCAGCAACCACA	255
*Cripto* (RT)	NM_011562	CTTTAAGCAGGGAGGTGGTG	TAAAGCCATCTGCCACAATG	195
*Rex-1* (RT)	NM_009556	ACATCCTAACCCACGCAAAG	TGATTTTCTGCCGTATGCAA	294
*Nestin* (R-T)	NM_016701	TAGAGGTGCAGCAGCTGCAG	AGCGATCTGACTCTGTAGAC	170
*Smooth muscle actin* (R-T)	NM_007392	ACTGGGACGACATGGAAAAG	CATCTCCAGAGTCCAGCACA	240
*Alpha fetoprotein* (R-T)	NM_007423	TGCACGAAAATGAGTTTGGGA	TTGCAGCCAACACATCGCTA	159
*Pax6* (R-T) [13]	NM_001244201	AGTTCTTCGCAACCTGGCTA	GTGTTCTCTCCCCCTCCTTC	186
*Kdr* (R-T) [14]	NM_010612	GGCGGTGGTGACAGTATCTT	CTCGGTGATGTACACGATGC	189
*Bmp1* (R-T)	NM_009755	TGAGGTGAATGGGGTGAAGC	TGTGGGCAGAGTAGCCATTG	167
*Ngf* (R-T)	NM_013609	GTGAACATGCTGTGCCTCAAG	GCGGCCAGTATAGAAAGCTG	186
*Tgfb3* (R-T)	NM_009368	GATCACCACAACCCACACCT	AGGTTCGTGGACCCATTTCC	195
*Wnt5a* (R-T)	NM_009524	TGACAATCAGGAGGCGTGAG	GGGCGTGATTGTGCAAAAGA	197
*Wnt8a* (R-T)	NM_009290	GGGAACGGTGGAATTGTCC	CAGCCGCAGTTTTCCAAGTC	163

RT, reverse-transcriptase PCR; R-T, real-time PCR.

For *in vitro* differentiation, parental ESCs and neoplastic tissue-derived ESC-like cells were cultured in LIF-free medium to induce embryoid body (EB) formation. On day 7 of culture, EBs were retrieved, and the expression levels of α-fetoprotein (*AFP*; endodermal), *nestin* (ectodermal), *Pax6* (ectodermal), *Kdr* (mesodermal), and smooth muscle actin (*SMA*; mesodermal) were monitored using quantitative PCR.

To evaluate chromosomal normality in ESCs and ESC-like cells, karyotype analysis was performed on passage 20 cell culture. The cells were collected by trypsinization and then suspended in hypotonic 0.075 M KCl solution (Sigma-Aldrich) at 37°C for 15 min. They were subsequently fixed in a 3∶1 mixture of methanol and acetic acid (both Sigma-Aldrich). Chromosome spreads were produced by dropping the solution onto slides followed by staining with Giemsa solution (GIBCO Invitrogen). The number of chromosomes within a cell was counted visually under a microscope. At least 17 cells in metaphase were counted in each group, and cells harboring 40 chromosomes were regarded as normal.

### DNA microsatellite analysis

To perform microsatellite analysis of the re-expanded ESC-like cells, genomic DNA was extracted using an AccuPrep Genomic DNA Extraction kit (Bioneer) following the manufacturer's instructions. Three specific mouse microsatellite primers (D15Mit159, D3Mit200, and D11Mit4) were obtained from a public database (Center for Inherited Disease Research [CIDR], Mouse Marker Set) and used to PCR-amplify the extracted genomic DNA at three microsatellite loci. The PCR products were size-fractioned on 3% agarose gels and visualized using EtBr staining.

### Analysis of DNA microarrays

Affymetrix chip data from the samples were generated from DNALINK incorporation (Seoul, Korea) according to standard protocols for microarray data analysis. After hybridization and staining of the arrays (Affymetrix GeneChip Mouse Gene 1.0 ST Array), images were acquired using an Affymetrix GeneChip Scanner 3000 7G, and the intensity obtained from each probe was extracted using Affymetrix GCOS software. All data were processed using a robust multi-array average (RMA) algorithm. The expression values were computed from CEL files by the applying RMA model of the probe-specific correction of perfect-match probes. These corrected values were then normalized via quantile normalization, and the resultant expression values were log_2_ transformed. The genes exhibiting a more than two-fold change in expression relative to the control were defined as differentially expressed genes (DEGs). Gene ontology (GO) annotation for DEGs was performed subsequently using DAVID bioinformatics resources (http://david.abcc.ncifcrf.gov; Tables S1–S7 in File S1). The microarray data were deposited in NCBI's Gene Expression Omnibus (GEO Series accession number: GSE58725).

### Quantitative PCR

To compare the relative mRNA levels in samples, cDNA synthesized from total RNA was quantified using real-time PCR and a DyNAmo SYBR Green qPCR Kit (Finnzymes, Espoo, Finland). The mRNA level of each gene was normalized to that of *β-actin* and defined as 2^-ΔΔCt^, where Ct  =  the threshold cycle for target amplification, ΔCt  =  Ct_target gene_ – Ct_internal reference (*β-actin*)_, and ΔΔCt  =  ΔCt_sample_ – ΔCt_calibrator_.

### Statistical analyses

A generalized linear model (PROC-GLM) in the Statistical Analysis System (SAS) program was used for statistical analysis. When analysis of variance (ANOVA) in the SAS package detected a significant main effect, the least squares method was conducted. Significant differences among treatments were defined as *p*<0.05.

## Results

### ESC injection with or without somatic Cells

A total of 19 injections were performed: four controls (vehicle and homologous somatic cell injection without ESCs), four single ESC line injections into homologous recipients, seven single ESC line injections into heterologous recipients, and four ESC and somatic cell co-injections into heterologous recipients. As shown in Table 2, a significant model effect was observed for teratoma formation after the cell injections (*p* = 0.0034). Control treatment did not induce teratoma formation, whereas all homologous injections (*n* = 4) induced formation. This activity was decreased significantly (100 vs. 14%; *p*<0.05) after heterologous injection without the co-injection of somatic cells. However, heterologous treatment with the co-injection of somatic cells re-established teratoma formation (75%; *p*<0.05 vs. heterologous injection without somatic cells).

**Table 2:** Teratoma formation upon subcutaneous injection of embryonic stem cells (ESC) with or without somatic cells.

Types of treatments		Type of transplantation	Genotype		Recipient strains	No. of injections	No. (%)^b^ of	
Stem cell injection^a^	Co-injected cell		ESC	Co-injected cell			Teratoma formed	ESC-like colonies repopulated^c^
No	None (vehicle)	N/A	N/A	None	B6D2F1	2	0 (0)^d^	N/A
No	Somatic cells (fetal fibroblasts)	N/A	N/A	B6D2F1	B6D2F1	2	0 (0)^d^	N/A
Yes	None	Homologous	B6CBAF1	None	B6CBAF1	4	4 (100)^e^	4 (100)^d^
Yes	None	Heterologous	B6CBAF1	None	B6D2F1	7	1 (14)^d^	1 (14)^e^
Yes	Somatic cells (fetal fibroblast)	Heterologous	B6CBAF1	B6D2F1	B6D2F1	4	3 (75)^e^	3 (75)^d^

Model effect of treatments in teratoma formation and ESC-like colony repopulated was 0.0034 and 0.0064, respectively (p value).

a Total 1×10^7^ cells with 1∶4 ratio of fetal fibroblasts and ESC in 100 µl PBS were injected.

b Percentage of the number of injections.

c Only three groups (homologous and heterologous injections) were statistically compared.

de Different superscripts indicate significant difference among the treatments, p<0.05.

### Repopulation and characterization of ESC-like cells

**Figure 1:**
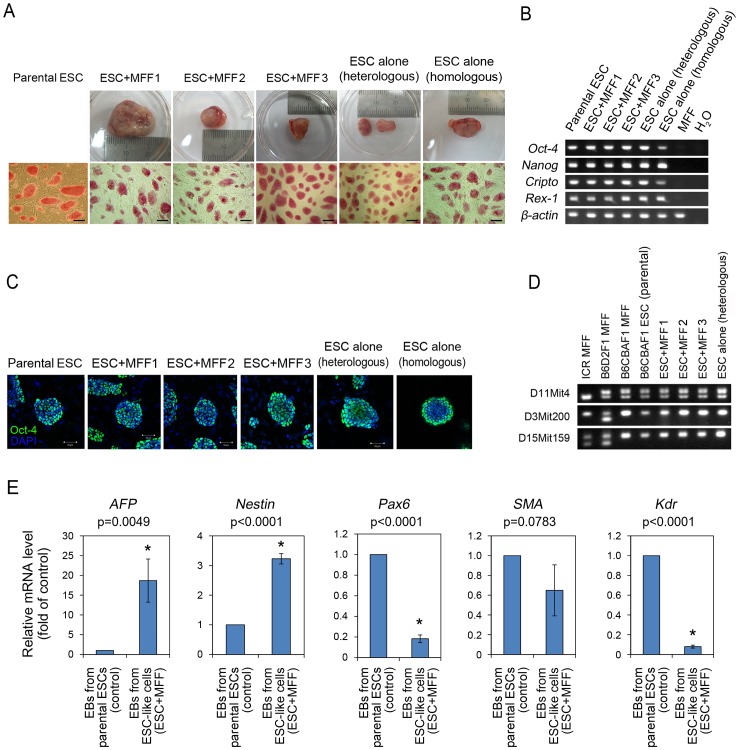
Establishment of embryonic stem cell (ESC)-like cells by the culture of teratomas derived from the co-injection of ESC and somatic cells. (A) Image of teratomas and colony-forming, ESC-like cells after alkaline phosphatase (AP) staining (B) Identification of stem cell-specific *Oct-4*, *Nanog*, *Cripto* and *Rex-1* gene expression and (C) Oct-4 translation by RT-PCR and immunostaining (scale bar  = 50 µm). All ESC-like cell colonies established showed similar morphology, AP activity and expression level of ESC-specific genes and protein with parental ESCs. (D) Short tandem repeat microsatellite analysis of established ESC-like cell colonies. All teratoma-derived, ESC-like cell lines were genetically matched with parental ESCs. (E) Germinal layer-specific gene expression in embryoid bodies (EBs) derived from parental ESCs and teratoma-derived ESC-like cells. The EBs from ESC-like cells showed the significant differences in *AFP*, *Nestin*, *Pax6*, and *Kdr* gene expression while they did not show the difference in *SMA* gene expression compared to the EBs derived from parental ESCs. ANOVA-DUNCAN was employed for statistical analysis and asterisks indicate significant differences.

Three groups of homologous and heterologous injections yielded eight teratomas, which all formed ESC-like colonies after culturing; there was a significant model effect among the three groups (Table 2, *p* = 0.0064). A sufficient number of colonies were obtained from all groups to allow establishment of an ESC-like cell line. All colonies derived from the teratomas had ESC-like characteristics including morphology, alkaline phosphatase activity (Fig. 1A), and expression of stemness-related genes and proteins, such as *Oct-4*, *Nanog*, *Cripto*, and *Rex-1* genes (Fig. 1B) and Oct-4 protein (Fig. 1C). A short tandem repeat microsatellite analysis confirmed the genetic matching of ESC-like cells with parental ESCs (Fig. 1D). Both parental ESCs and ESC-like cells repopulated after co-injection of ESCs with MFF formed embryoid bodies (EBs); however, the two EB populations coexpressed different levels of three germ layer-specific genes (Fig. 1E). Significantly elevated expression of *AFP* (endodermal) and *Nestin* (ectodermal) was detected in ESC-like cells relative to parental ESCs, whereas the expression of *Pax6* (ectodermal) and *Kdr* (mesodermal) was reduced significantly in ESC-like cells. No difference in the expression of *SMA* (mesodermal) was detected.

**Figure 2:**
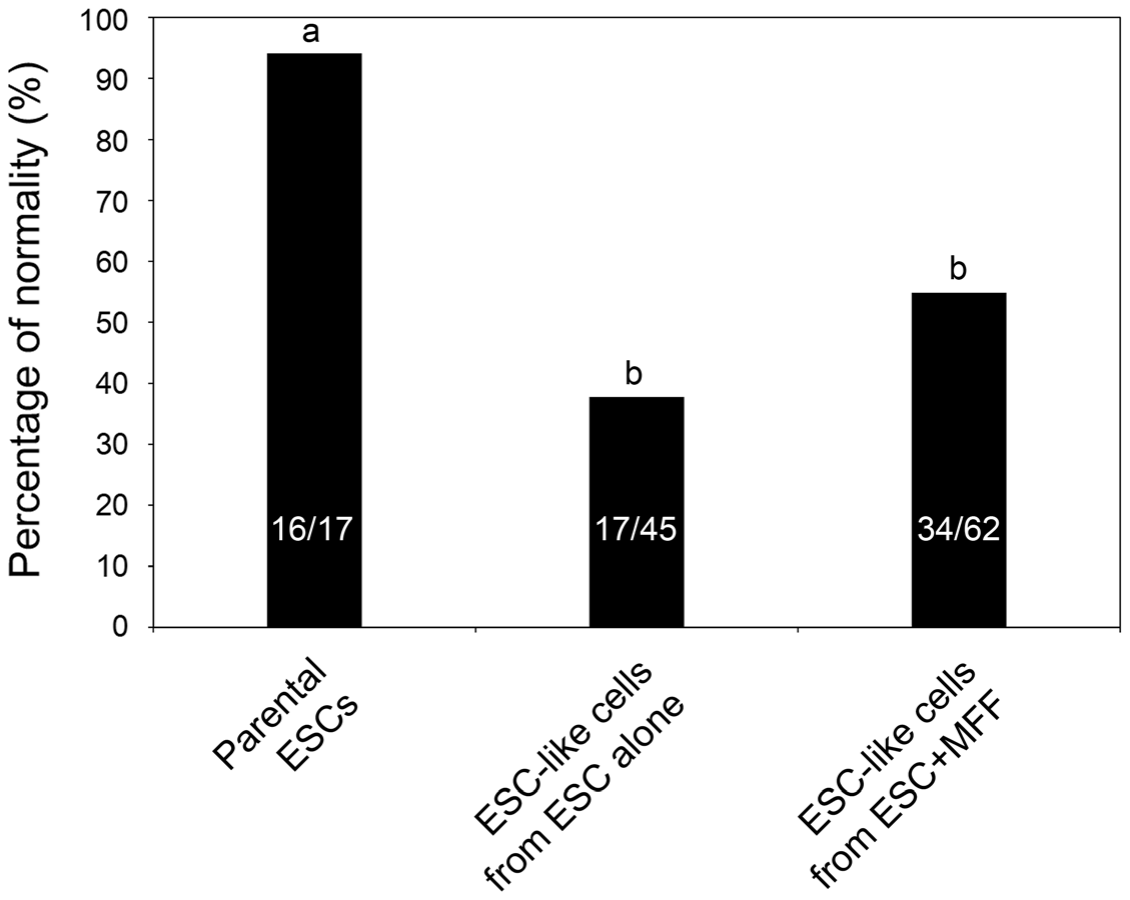
Chromosome normality of repopulated embryonic stem cell (ESC)-like cells after co-injection of ESCs and somatic cells. The normality was compared between parental ESCs and ESC-like cells repopulated from teratomas. PROC-GLM in SAS detected remarkable decreases of chromosome normality in all ESC-like cells repopulated compared to parental ESCs (p = 0.0003). The numbers within bars indicate the number of normal cells/the number of total cells counted. Different letters^a,b^ indicate significant difference among the groups, p<0.05.

As shown in Fig. 2, karyotype analysis demonstrated a significant model effect of chromosome normality between parental ESCs and repopulated ESC-like cells (*p* = 0.0003). Compared with the 94% normal diploid chromosome number in parental ESCs, a sharp increase in aneuploidy was detected in repopulated ESC-like cells, regardless of co-injection with somatic cells (38 to 55%; *p*<0.05).

### Gene expression profiling

**Figure 3:**
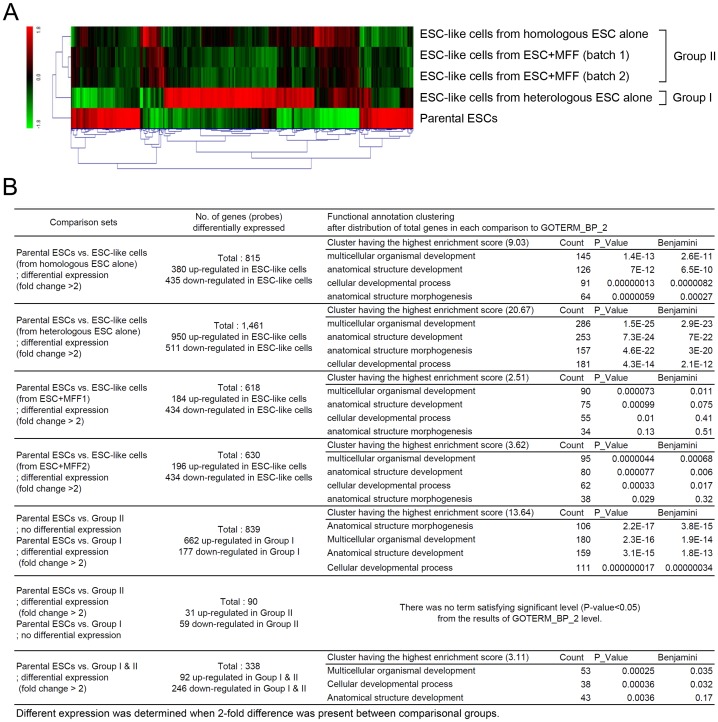
Clustering analysis and gene ontology annotation. (A) Clustering analysis was designed between parental embryonic stem cells (ESCs) and ESC-like cell lines repopulated by the culture of the teratomas following ESC injection. A heatmap for all samples was established and based on clustering result, four lines of repopulated ESC-like cells were allotted into two distinct groups according to their expression patterns. (B) From the comparisons between parental ESCs and ESC-like cells from various conditions and between parental ESCs and clustered groups, differentially expressed probes were derived. The probe sets derived from each comparison were distributed into various gene ontology terms on biological function in level 2 when analyzed by DAVID gene annotation tool. Obtained data were further analyzed by functional annotation clustering (Full gene ontology terms derived from each comparison set were listed in Table S1-S7 in File S1).

Gene expression profiles were generated from five cell populations: parental ESCs and four ESC-like cell populations (homologous ESC alone, heterologous ESC alone, ESCs with MFF batch 1, and ESCs with MFF batch 2). Clustering analysis revealed 1,856 differentially-expressed probes (DEPs; defined as >2-fold change) among the cell populations (Fig. 3A). A comparison between the parental ESCs and ESC-like cells repopulated from the homologous injection of a single ESC line (Figure 3B) revealed 815 DEPs, of which 380 and 435 were up- and down-regulated in the repopulated cells, respectively. In contrast, the number of DEPs between the parental ESCs and ESC-like cells repopulated from the heterologous injection of a single ESC line was 1,461. Fewer DEPs were detected in the comparison between parental ESCs and ESC-like cell lines derived from co-injections (618 and 630 in batch 1 and 2, respectively). When the four groups of DEPs derived from each comparison were subjected to functional annotation clustering analysis, the DEPs generated four major clusters: multicellular organismal development, anatomical structure development, cellular developmental process, and anatomical structural morphogenesis.

To identify the factors that influenced the differences in the number of DEPs among groups, additional comparison sets were designed based on three different expression clusters: parental ESCs, ESC-like cells repopulated after heterologous injection of ESCs alone (designated Group I), and ESC-like cells repopulated after homologous injection of ESCs alone and heterologous co-injection of ESCs and somatic cells (designated Group II). As shown in Fig. 3B, 839 probe sets were differentially expressed between the parental ESCs and Group I, but not between the parental ESCs and Group II. Ninety probe sets revealed differential expression between the parental ESCs and Group II, but not between the parental ESCs and Group I, and 338 probe sets showed differential expression between the parental ESCs and both Group I and II. DAVID gene annotation demonstrated that among the DEPs represented by the 839 probe sets, the cluster with the highest enrichment score (13.64) included four GO terms: anatomical structure morphogenesis, multicellular organismal development, anatomical structure development, and cellular developmental process (*p*<0.05). Among the 90 probe sets identified, there was no significant distribution of probes into specific terms in GOTERM_BP_2 level, whereas with the group of 338 probe sets, the cluster with the highest enrichment score (3.11) included multicellular organismal development and cellular developmental process (*p*<0.05).

**Figure 4:**
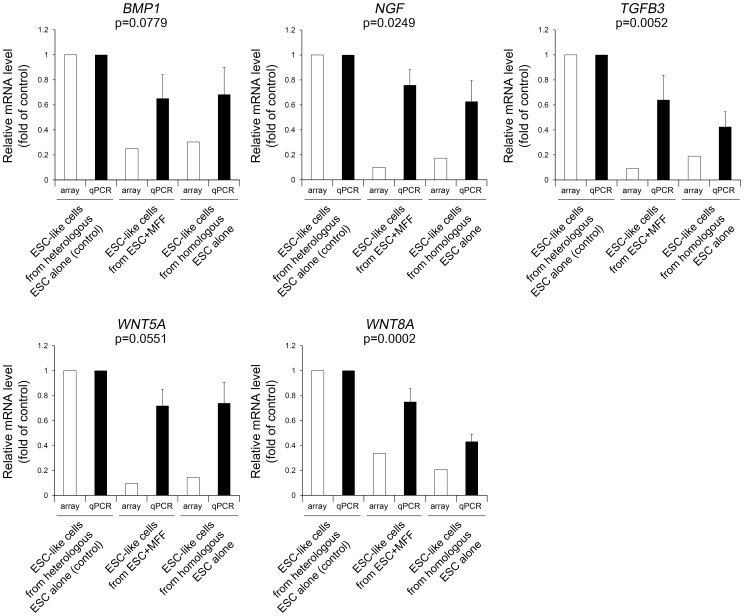
Quantification of mRNA expression. Five pluripotency-regulatory genes including *BMP1, NGF, TGFB3, WNT5A, and WNT8A* were selected from 839 probes showing both differential expression between parental ESCs and Group I and no differential expression between parental ESCs and Group II on the basis of microarray results. Three different ESC-like cells derived from the injection of heterologous ESCs alone, somatic cell co-injection with heterologous ESCs, and homologous ESCs alone were provided for quantitative RT-PCR analysis. For side by side comparison, the results were presented along with microarray results corresponding to each of genes. Similar gene expression pattern to microarray was detected in all five genes analyzed. Statistical analysis was performed by ANOVA-DUNCAN in SAS program. The p value in each graph indicates model effect among quantitative RT-PCR levels.

To validate the microarray results, five pluripotency-regulatory genes (*BMP1, NGF, TGFB3, WNT5A, and WNT8A*) were selected from the 839 probes identified in the first comparison. Their expression was then assessed using quantitative RT-PCR in three different ESC-like cell lines that repopulated after heterologous injection of ESCs alone, heterologous co-injection of ESCs and somatic cells, and homologous injection of ESCs alone. All five genes showed similar gene expression patterns to those identified by microarray analysis. As shown in Fig. 4, a significant model effect was detected in three of the comparisons (*NGF*, *TGFB3*, and *WNT8A*), whereas the other two comparisons (*BMP1* and *WNT5A*) tended to differ significantly (*p*<0.08).

## Discussion

The initial aim of this study was to monitor various bio-reactions triggered by stem cell transplantation and to subsequently evaluate whether the co-injection of somatic cells with ESCs could provide contextual cues to optimize the growth and differentiation of transplanted stem cells. Based on the biological events that occurred after transplantation, the primary aim then was to examine the effects of co-injection of somatic cells with ESCs on cell engraftment. The results demonstrated that the co-injection of somatic cells with ESCs influenced host-to-cell adaptability, post-injection cell transformation and gene expression in re-expanded stem cell-like cells derived from neoplastic tissues. Finally, we examined whether transplanted cells could act as progenitors of neoplastic tissues. The results demonstrated that the expansion of aneuploid stem cell-like cells might affect the future establishment of stem cell-derived cancer stem cells, although future studies are needed for definitive conclusions.

The results of this study demonstrated that 1) homogeneity between the donor and recipient plays a critical role in stem cell activity after injection, and 2) the exposure of stem cells to recipient cells before injection alleviates the donor-host interaction. The exposure of ESCs to somatic cells homologous with the recipients alters teratoma formation *in vivo* and modifies gene function. Gene expression in the repopulated ESC-like cells was also influenced by this exposure, although the genetic instability primarily caused by stem cell repopulation could not be recovered by co-injection with somatic cells. This suggests that both favorable and adverse effects could be triggered in transplanted stem cells by exposure to syngeneic somatic cells during the cell manipulation procedure. The appropriate selection and use of the co-injected somatic cells might help regulate stem cell function. From a clinical viewpoint, our findings suggest a potential way to regulate differentiation activity *in vivo*, which differs from differentiation *in vitro*. Nevertheless, the detailed actions of co-injected somatic cells remain unclear. It is possible that the simple exposure of stem cells to the host phenotype and the gene expression profile are insufficient to fully explain the effects of co-injected somatic cells observed in the current study. The nature of the somatic cells themselves might also affect the properties of the transplanted stem cells.

A number of somatic cells such as bone marrow cells and adult human fibroblasts have immunosuppressive properties [10], [11]. The co-injection of stem cells with somatic cells may alter the mechanism of host defense against the injected stem cells by inducing both genetic and epigenetic changes. The results of this study suggest that the co-injection of somatic cells decreased the difference in gene expression between parental ESCs and repopulated ESC-like cells. This suggests that the post-injection activity of ESCs in heterologous recipients is affected by the co-injected somatic cells, which provides useful information for the clinical application of stem cell-based therapy. Aneuploidy is increased markedly in teratoma-derived repopulated ESCs. Incomplete reprogramming or self-renewal under a teratoma niche created after ESC transplantation might cause aneuploidy. A heterologous population of ESCs and other aneuploid tumor cells might also explain these results. Regardless of the reason, the niche created after the injection of stem cells might induce genetic plasticity due to incomplete reprogramming and/or self-renewal. It might also represent the genetic plasticity of either the cancer stem cells or tumor-derived stem cells.

A critical marker demonstrating the pluripotency of ESCs is their ability to form teratoma, which suggests that transplanted ESCs have the ability to differentiate [12]. As such, the expression pattern of development-related genes in the transplanted ESCs was altered by the formation of teratoma. Our results further suggest that the co-injection of somatic cells with ESCs alters the transcription of genes related to anatomical structural morphogenesis, mutilcellular organismal development, anatomical structure development and cellular developmental process. It is possible that co-injection with somatic cells might modulate ESC differentiation *in vivo*. Therefore, the use of appropriate somatic cells during the heterologous transplantation of ESCs might contribute to the regulation of target differentiation and lead to successful cell replacement therapy. However, some genes in the clusters showed *de novo* altered gene expression, regardless of co-injection with somatic cells.

The data from the current study suggest that homologous components (homologous ESCs themselves or co-injection with homologous somatic cells) play crucial roles in ESC differentiation *in vivo*. This highlights the potential importance of genetic compatibility in ESC plasticity after transplantation. However, the re-population of stem cell-like cells in the current study might induce chromosomal instability.

In conclusion, the current study introduces a novel model for understanding stem cell transformation after cell transplantation. A mouse model of homologous or heterologous stem cell transplantation could be used to develop novel techniques for tissue regeneration and regulating differentiation. The use of the simultaneous injection of somatic cells with ESCs might be useful in heterologous recipients and might reduce the risk of unexpected differentiation. Further studies on cell transformation will help distinguish tumorigenic from undifferentiated cells, as well as establish stem cell differentiation in the host.

## Supporting Information

File S1 **Tables S1-S7.**
(DOCX)
