# Supplementary material for: The Co-Injection of Somatic Cells with Embryonic Stem Cells Affects Teratoma Formation and the Properties of Teratoma-Derived Stem Cell-Like Cells
Source: PLoS One. 2014 Sep 2;9(9):e105975. doi: 10.1371/journal.pone.0105975 (PMC4152121; doi:10.1371/journal.pone.0105975)
Supplement: File S1 — Tables S1-S7. (DOCX) [file pone.0105975.s001.docx]

**Table S1.** Gene ontology terms on biological process in level 2 by gene ontology annotation analysis of 815 probe sets that showed differential expression between parental ESCs and ESC-like cells from homologous ESC injection alone.

| Term | Count | % | P-Value | Benjamini |
| --- | --- | --- | --- | --- |
| multicellular organismal development | 145 | 21.4 | 1.4E-13 | 2.6E-11 |
| anatomical structure development | 126 | 18.6 | 7E-12 | 6.4E-10 |
| cellular developmental process | 91 | 13.4 | 1.3E-07 | 8.1E-06 |
| anatomical structure morphogenesis | 64 | 9.4 | 5.9E-06 | 0.00027 |
| negative regulation of cellular process | 62 | 9.1 | 0.00015 | 0.0057 |
| negative regulation of biological process | 67 | 9.9 | 0.00018 | 0.0056 |
| regulation of multicellular organismal process | 44 | 6.5 | 0.00032 | 0.0084 |
| membrane organization | 21 | 3.1 | 0.00048 | 0.011 |
| regulation of developmental process | 33 | 4.9 | 0.0014 | 0.028 |
| negative regulation of developmental process | 17 | 2.5 | 0.0025 | 0.045 |
| positive regulation of biological process | 68 | 10 | 0.0025 | 0.042 |
| positive regulation of cellular process | 60 | 8.8 | 0.0052 | 0.077 |
| ossification | 10 | 1.5 | 0.0069 | 0.092 |
| cell projection organization | 20 | 2.9 | 0.0071 | 0.089 |
| regulation of metabolic process | 116 | 17.1 | 0.0091 | 0.11 |
| embryonic development | 33 | 4.9 | 0.016 | 0.17 |
| antigen processing and presentation | 8 | 1.2 | 0.021 | 0.21 |
| alcohol metabolic process | 20 | 2.9 | 0.023 | 0.21 |
| regulation of cellular component biogenesis | 8 | 1.2 | 0.024 | 0.21 |
| cellular component morphogenesis | 19 | 2.8 | 0.034 | 0.27 |
| synaptonemal complex organization | 3 | 0.4 | 0.039 | 0.29 |
| negative regulation of multicellular organismal process | 8 | 1.2 | 0.041 | 0.3 |
| positive regulation of cellular component organization | 9 | 1.3 | 0.042 | 0.29 |
| anatomical structure formation involved in morphogenesis | 19 | 2.8 | 0.046 | 0.3 |
| reproductive cellular process | 11 | 1.6 | 0.051 | 0.32 |
| response to chemical stimulus | 38 | 5.6 | 0.058 | 0.34 |
| cell death | 24 | 3.5 | 0.059 | 0.34 |
| reproductive process | 28 | 4.1 | 0.064 | 0.35 |
| organic ether metabolic process | 5 | 0.7 | 0.067 | 0.35 |
| vesicle-mediated transport | 22 | 3.2 | 0.072 | 0.37 |
| regulation of molecular function | 25 | 3.7 | 0.073 | 0.36 |
| reproductive developmental process | 14 | 2.1 | 0.082 | 0.39 |
| cell motion | 18 | 2.7 | 0.083 | 0.38 |
| positive regulation of developmental process | 12 | 1.8 | 0.083 | 0.37 |
| fertilization | 6 | 0.9 | 0.084 | 0.37 |
| negative regulation of immune system process | 6 | 0.9 | 0.096 | 0.4 |

**Table S2.** Gene ontology terms on biological process in level 2 by gene ontology annotation analysis of 1,461 probe sets that showed differential expression between parental ESCs and ESC-like cells from heterologous ESC injection alone.

| Term | Count | % | P-Value | Benjamini |
| --- | --- | --- | --- | --- |
| multicellular organismal development | 286 | 22.2 | 1.5E-25 | 2.9E-23 |
| anatomical structure development | 253 | 19.7 | 7.3E-24 | 6.9E-22 |
| anatomical structure morphogenesis | 157 | 12.2 | 4.6E-22 | 2.9E-20 |
| cellular developmental process | 181 | 14.1 | 4.3E-14 | 2.1E-12 |
| negative regulation of developmental process | 47 | 3.7 | 1.5E-12 | 5.9E-11 |
| regulation of developmental process | 81 | 6.3 | 1.4E-11 | 4.3E-10 |
| regulation of multicellular organismal process | 100 | 7.8 | 2E-11 | 5.5E-10 |
| positive regulation of biological process | 160 | 12.4 | 2.6E-11 | 6.2E-10 |
| negative regulation of biological process | 146 | 11.3 | 3.5E-11 | 7.4E-10 |
| positive regulation of cellular process | 144 | 11.2 | 1E-10 | 1.9E-09 |
| negative regulation of cellular process | 131 | 10.2 | 5.2E-10 | 9E-09 |
| embryonic development | 85 | 6.6 | 1.6E-09 | 2.5E-08 |
| cell adhesion | 74 | 5.7 | 4.1E-09 | 6E-08 |
| anatomical structure formation involved in morphogenesis | 54 | 4.2 | 1.6E-08 | 2.1E-07 |
| developmental growth | 23 | 1.8 | 2.6E-07 | 3.3E-06 |
| pattern specification process | 43 | 3.3 | 3E-07 | 3.5E-06 |
| extracellular structure organization | 28 | 2.2 | 7.6E-07 | 8.5E-06 |
| response to external stimulus | 73 | 5.7 | 1.7E-06 | 0.000019 |
| ossification | 22 | 1.7 | 2.9E-06 | 0.000029 |
| regulation of growth | 37 | 2.9 | 6.8E-06 | 0.000065 |
| cell motion | 44 | 3.4 | 0.000088 | 0.0008 |
| positive regulation of metabolic process | 70 | 5.4 | 0.0001 | 0.00086 |
| positive regulation of developmental process | 30 | 2.3 | 0.0001 | 0.00084 |
| membrane organization | 35 | 2.7 | 0.00014 | 0.0011 |
| cell growth | 10 | 0.8 | 0.00042 | 0.0032 |
| response to chemical stimulus | 83 | 6.4 | 0.00043 | 0.0032 |
| regulation of locomotion | 18 | 1.4 | 0.0006 | 0.0042 |
| tissue remodeling | 11 | 0.9 | 0.00065 | 0.0044 |
| response to endogenous stimulus | 25 | 1.9 | 0.00068 | 0.0045 |
| response to stress | 104 | 8.1 | 0.00072 | 0.0046 |
| negative regulation of cellular component organization | 16 | 1.2 | 0.00078 | 0.0048 |
| reproductive developmental process | 32 | 2.5 | 0.00079 | 0.0047 |
| cellular component morphogenesis | 39 | 3 | 0.001 | 0.006 |
| localization of cell | 33 | 2.6 | 0.0013 | 0.0074 |
| cell motility | 33 | 2.6 | 0.0013 | 0.0074 |
| actin filament-based process | 22 | 1.7 | 0.0043 | 0.023 |
| regulation of localization | 41 | 3.2 | 0.0044 | 0.023 |
| cell proliferation | 28 | 2.2 | 0.0045 | 0.023 |
| multicellular organismal metabolic process | 7 | 0.5 | 0.0051 | 0.026 |
| vesicle-mediated transport | 45 | 3.5 | 0.0063 | 0.031 |
| regulation of cellular component organization | 34 | 2.6 | 0.0074 | 0.035 |
| positive regulation of multicellular organismal process | 20 | 1.6 | 0.0081 | 0.037 |
| positive regulation of growth | 10 | 0.8 | 0.0086 | 0.039 |
| negative regulation of immune system process | 12 | 0.9 | 0.0087 | 0.038 |
| developmental maturation | 14 | 1.1 | 0.012 | 0.051 |
| regulation of response to stimulus | 33 | 2.6 | 0.013 | 0.053 |
| positive regulation of locomotion | 8 | 0.6 | 0.021 | 0.083 |
| taxis | 14 | 1.1 | 0.022 | 0.085 |
| synaptonemal complex organization | 4 | 0.3 | 0.022 | 0.086 |
| positive regulation of immune system process | 22 | 1.7 | 0.023 | 0.087 |
| regulation of metabolic process | 213 | 16.6 | 0.026 | 0.095 |
| regulation of cellular component biogenesis | 12 | 0.9 | 0.026 | 0.095 |
| anatomical structure arrangement | 3 | 0.2 | 0.036 | 0.13 |
| regulation of immune system process | 30 | 2.3 | 0.04 | 0.14 |
| regulation of biological quality | 87 | 6.8 | 0.043 | 0.15 |
| molting cycle | 8 | 0.6 | 0.046 | 0.15 |
| stem cell maintenance | 5 | 0.4 | 0.048 | 0.16 |
| reproductive cellular process | 18 | 1.4 | 0.051 | 0.16 |
| reproductive process | 51 | 4 | 0.054 | 0.17 |
| negative regulation of multicellular organismal process | 12 | 0.9 | 0.054 | 0.17 |
| negative regulation of response to stimulus | 9 | 0.7 | 0.054 | 0.16 |
| cell projection organization | 29 | 2.3 | 0.057 | 0.17 |
| negative regulation of homeostatic process | 3 | 0.2 | 0.069 | 0.2 |
| cell death | 42 | 3.3 | 0.073 | 0.21 |
| coagulation | 9 | 0.7 | 0.077 | 0.21 |
| ovulation cycle process | 7 | 0.5 | 0.078 | 0.21 |
| synaptogenesis | 4 | 0.3 | 0.078 | 0.21 |
| negative regulation of growth | 9 | 0.7 | 0.083 | 0.22 |
| response to abiotic stimulus | 23 | 1.8 | 0.083 | 0.22 |
| ovulation cycle | 7 | 0.5 | 0.084 | 0.22 |
| regulation of homeostatic process | 8 | 0.6 | 0.087 | 0.22 |
| positive regulation of cellular component organization | 13 | 1 | 0.09 | 0.22 |
| behavior | 34 | 2.6 | 0.093 | 0.23 |

**Table S3.** Gene ontology terms on biological process in level 2 by gene ontology annotation analysis of 618 probe sets that showed differential expression between parental ESCs and ESC-like cells from co-injection of ESCs and MFF batch 1.

| Term | Count | % | P-Value | Benjamini |
| --- | --- | --- | --- | --- |
| multicellular organismal development | 90 | 18 | 0.000073 | 0.011 |
| anatomical structure development | 75 | 15 | 0.00099 | 0.075 |
| cellular developmental process | 55 | 11 | 0.01 | 0.41 |
| Ossification | 8 | 1.6 | 0.014 | 0.43 |
| regulation of cellular component biogenesis | 7 | 1.4 | 0.021 | 0.48 |
| negative regulation of developmental process | 12 | 2.4 | 0.022 | 0.43 |
| synaptonemal complex organization | 3 | 0.6 | 0.023 | 0.41 |
| membrane organization | 13 | 2.6 | 0.033 | 0.48 |
| positive regulation of cellular process | 43 | 8.6 | 0.039 | 0.5 |
| positive regulation of growth | 5 | 1 | 0.046 | 0.52 |
| positive regulation of biological process | 47 | 9.4 | 0.047 | 0.5 |
| taxis | 7 | 1.4 | 0.048 | 0.48 |
| alcohol metabolic process | 15 | 3 | 0.054 | 0.49 |
| regulation of multicellular organismal process | 27 | 5.4 | 0.058 | 0.49 |
| regulation of developmental process | 21 | 4.2 | 0.058 | 0.47 |
| translational initiation | 4 | 0.8 | 0.063 | 0.47 |
| extracellular structure organization | 8 | 1.6 | 0.069 | 0.48 |
| negative regulation of cellular component organization | 6 | 1.2 | 0.074 | 0.49 |
| negative regulation of biological process | 41 | 8.2 | 0.082 | 0.51 |
| cell adhesion | 20 | 4 | 0.086 | 0.51 |
| positive regulation of locomotion | 4 | 0.8 | 0.089 | 0.5 |
| regulation of growth | 11 | 2.2 | 0.092 | 0.5 |
| negative regulation of cellular process | 37 | 7.4 | 0.093 | 0.49 |
| fertilization | 5 | 1 | 0.1 | 0.5 |
| positive regulation of transport | 7 | 1.4 | 0.1 | 0.48 |

**Table S4.** Gene ontology terms on biological process in level 2 by gene ontology annotation analysis of 630 probe sets that showed differential expression between parental ESCs and ESC-like cells from co-injection of ESCs and MFF batch 2.

| Term | Count | % | P-Value | Benjamini |
| --- | --- | --- | --- | --- |
| multicellular organismal development | 95 | 19.1 | 4.4E-06 | 0.00068 |
| anatomical structure development | 80 | 16.1 | 0.000077 | 0.006 |
| cellular developmental process | 62 | 12.5 | 0.00033 | 0.017 |
| positive regulation of cellular process | 48 | 9.7 | 0.0043 | 0.15 |
| positive regulation of biological process | 51 | 10.3 | 0.0099 | 0.27 |
| ossification | 8 | 1.6 | 0.014 | 0.31 |
| membrane organization | 14 | 2.8 | 0.015 | 0.29 |
| negative regulation of biological process | 45 | 9.1 | 0.018 | 0.3 |
| regulation of developmental process | 23 | 4.6 | 0.02 | 0.29 |
| negative regulation of developmental process | 12 | 2.4 | 0.022 | 0.29 |
| synaptonemal complex organization | 3 | 0.6 | 0.023 | 0.28 |
| anatomical structure morphogenesis | 38 | 7.6 | 0.029 | 0.32 |
| negative regulation of cellular process | 40 | 8 | 0.031 | 0.31 |
| regulation of multicellular organismal process | 28 | 5.6 | 0.036 | 0.34 |
| taxis | 7 | 1.4 | 0.048 | 0.4 |
| reproductive cellular process | 9 | 1.8 | 0.059 | 0.44 |
| translational initiation | 4 | 0.8 | 0.063 | 0.45 |
| regulation of cellular component biogenesis | 6 | 1.2 | 0.064 | 0.44 |
| positive regulation of cellular component organization | 7 | 1.4 | 0.075 | 0.47 |
| positive regulation of locomotion | 4 | 0.8 | 0.089 | 0.52 |
| embryonic development | 23 | 4.6 | 0.089 | 0.5 |
| regulation of growth | 11 | 2.2 | 0.092 | 0.49 |
| transport | 67 | 13.5 | 0.093 | 0.48 |
| regulation of metabolic process | 82 | 16.5 | 0.099 | 0.49 |
| positive regulation of transport | 7 | 1.4 | 0.1 | 0.48 |

**Table S5.** Gene ontology terms on biological process in level 2 by gene ontology annotation analysis of 839 probe sets that showed both differential expression between parental ESCs and Group I and no differential expression between parental ESCs and Group II.

| Terms | Count | % | P-Value | Benjamini |
| --- | --- | --- | --- | --- |
| anatomical structure morphogenesis | 106 | 13.5 | 2.2E-17 | 3.8E-15 |
| multicellular organismal development | 180 | 22.9 | 2.3E-16 | 1.9E-14 |
| anatomical structure development | 159 | 20.3 | 3.1E-15 | 1.8E-13 |
| cell adhesion | 56 | 7.1 | 5.8E-10 | 2.5E-08 |
| positive regulation of biological process | 106 | 13.5 | 3.6E-09 | 1.2E-07 |
| embryonic development | 60 | 7.6 | 8.1E-09 | 2.3E-07 |
| developmental growth | 20 | 2.5 | 1.3E-08 | 3.2E-07 |
| positive regulation of cellular process | 95 | 12.1 | 1.6E-08 | 3.4E-07 |
| cellular developmental process | 111 | 14.1 | 1.7E-08 | 3.4E-07 |
| regulation of developmental process | 52 | 6.6 | 4.8E-08 | 8.4E-07 |
| regulation of multicellular organismal process | 63 | 8 | 1.3E-07 | 2.1E-06 |
| anatomical structure formation involved in morphogenesis | 38 | 4.8 | 1.9E-07 | 2.7E-06 |
| negative regulation of developmental process | 28 | 3.6 | 3.6E-07 | 4.8E-06 |
| negative regulation of biological process | 88 | 11.2 | 2E-06 | 0.000025 |
| pattern specification process | 30 | 3.8 | 3.5E-06 | 0.00004 |
| negative regulation of cellular process | 80 | 10.2 | 4.4E-06 | 0.000048 |
| response to external stimulus | 50 | 6.4 | 8.9E-06 | 0.000091 |
| regulation of growth | 27 | 3.4 | 1.2E-05 | 0.00012 |
| extracellular structure organization | 19 | 2.4 | 2.7E-05 | 0.00025 |
| cell motility | 28 | 3.6 | 2.7E-05 | 0.00024 |
| localization of cell | 28 | 3.6 | 2.7E-05 | 0.00024 |
| cell motion | 33 | 4.2 | 2.9E-05 | 0.00024 |
| positive regulation of developmental process | 23 | 2.9 | 4.7E-05 | 0.00037 |
| ossification | 15 | 1.9 | 0.00008 | 0.0006 |
| response to stress | 72 | 9.2 | 0.00033 | 0.0024 |
| positive regulation of metabolic process | 45 | 5.7 | 0.0012 | 0.0081 |
| regulation of locomotion | 13 | 1.7 | 0.0015 | 0.0097 |
| actin filament-based process | 17 | 2.2 | 0.0018 | 0.012 |
| reproductive developmental process | 22 | 2.8 | 0.0021 | 0.013 |
| tissue remodeling | 8 | 1 | 0.0024 | 0.015 |
| response to chemical stimulus | 52 | 6.6 | 0.0053 | 0.031 |
| response to endogenous stimulus | 16 | 2 | 0.007 | 0.039 |
| cell proliferation | 19 | 2.4 | 0.01 | 0.055 |
| cellular component morphogenesis | 24 | 3.1 | 0.014 | 0.07 |
| cell growth | 6 | 0.8 | 0.015 | 0.075 |
| multicellular organismal metabolic process | 5 | 0.6 | 0.019 | 0.09 |
| coagulation | 8 | 1 | 0.021 | 0.099 |
| regulation of localization | 26 | 3.3 | 0.021 | 0.097 |
| positive regulation of growth | 7 | 0.9 | 0.024 | 0.1 |
| membrane organization | 19 | 2.4 | 0.025 | 0.11 |
| negative regulation of homeostatic process | 3 | 0.4 | 0.029 | 0.12 |
| positive regulation of locomotion | 6 | 0.8 | 0.03 | 0.12 |
| positive regulation of multicellular organismal process | 13 | 1.7 | 0.03 | 0.12 |
| negative regulation of cellular component organization | 9 | 1.1 | 0.032 | 0.12 |
| ovulation cycle process | 6 | 0.8 | 0.039 | 0.14 |
| ovulation cycle | 6 | 0.8 | 0.042 | 0.15 |
| regulation of cellular component organization | 21 | 2.7 | 0.042 | 0.15 |
| developmental maturation | 9 | 1.1 | 0.049 | 0.17 |
| vesicle-mediated transport | 27 | 3.4 | 0.054 | 0.18 |
| molting cycle | 6 | 0.8 | 0.056 | 0.18 |
| regulation of biological process | 281 | 35.8 | 0.057 | 0.18 |
| negative regulation of growth | 7 | 0.9 | 0.064 | 0.2 |
| regulation of body fluid levels | 8 | 1 | 0.065 | 0.2 |

**Table S6.** Gene ontology terms on biological process in level 2 by gene ontology annotation analysis 90 probe sets that showed both no differential expression between parental ESCs and Group I and differential expression between parental ESCs and Group II.

| Terms | Count | % | P-Value | Benjamini |
| --- | --- | --- | --- | --- |
| regulation of molecular function | 7 | 9.6 | 0.01 | 0.58 |
| regulation of metabolic process | 17 | 23.3 | 0.033 | 0.75 |
| positive regulation of biological process | 10 | 13.7 | 0.065 | 0.85 |
| positive regulation of transport | 3 | 4.1 | 0.075 | 0.81 |
| positive regulation of cellular process | 9 | 12.3 | 0.079 | 0.75 |

**Table S7.** Gene ontology terms on biological process in level 2 by gene ontology annotation analysis of 338 probe sets that showed differential expression between parental ESCs and all the other clusters.

| Terms | Count | % | P-Value | Benjamini |
| --- | --- | --- | --- | --- |
| multicellular organismal development | 53 | 19.3 | 0.00026 | 0.035 |
| cellular developmental process | 38 | 13.8 | 0.00047 | 0.032 |
| anatomical structure development | 43 | 15.6 | 0.0041 | 0.17 |
| synaptonemal complex organization | 3 | 1.1 | 0.007 | 0.22 |
| sexual reproduction | 12 | 4.4 | 0.012 | 0.28 |
| reproductive cellular process | 7 | 2.5 | 0.025 | 0.44 |
| regulation of cellular component biogenesis | 5 | 1.8 | 0.028 | 0.43 |
| ossification | 5 | 1.8 | 0.049 | 0.58 |
| membrane organization | 8 | 2.9 | 0.064 | 0.64 |
| gamete generation | 9 | 3.3 | 0.066 | 0.61 |
| fertilization | 4 | 1.5 | 0.069 | 0.59 |
| anatomical structure morphogenesis | 21 | 7.6 | 0.085 | 0.64 |
